# Revisiting cytomegalovirus in pediatric allogeneic hematopoietic stem cell transplant recipients: current strategies for prevention and management in the letermovir era

**DOI:** 10.3389/fped.2025.1663600

**Published:** 2025-09-12

**Authors:** Lisa Hiskey, Diego R. Hijano, Ramia Zakhour

**Affiliations:** ^1^ Department of Infectious Diseases, St. Jude Children’s Research Hospital, Memphis, TN, United States; ^2^ Department of Pediatrics, University of Tennessee Health Science Center, Memphis, TN, United States; ^3^ Division of Infectious Diseases, Department of Pediatrics, McGovern Medical Center at UTHealth Houston, Children’s Memorial Hermann Hospital, Houston, TX, United States

**Keywords:** CMV, antiviral, hematopoietic cell transplant, pediatric, letermovir

## Abstract

CMV infection remains the most common clinically significant infection in pediatric allogeneic hematopoietic stem cell (HCT) recipients, with seropositive recipients of transplants from seronegative donors at the highest risk for complications. In recent years, letermovir, a novel antiviral with a favorable toxicity profile, was approved first for adults and more recently for pediatric patients for the primary prophylaxis of CMV infection and disease in high-risk HCT recipients. Growing evidence from real-life data regarding the safety and efficacy of letermovir has transformed the landscape of CMV disease in HCT transplant recipients and led to a paradigm shift from a preemptive approach to prophylaxis. Other novel additions to the diagnosis, risk stratification, and treatment of CMV include the measurement of CMV-specific cellular-mediated immunity and the approval of maribavir as a treatment option for resistant/refractory CMV infection and disease. Other prevention and treatment modalities currently under development include virus-specific T cells, monoclonal antibodies, and vaccines. Despite these promising advancements, additional pediatric-specific studies are needed to better understand the viral and immunological implications of these novel preventive and therapeutic methods in this patient population.

## Highlights

Letermovir, a novel antiviral agent with a favorable toxicity profile, has transformed CMV disease management in hematopoietic stem cell transplant recipients, shifting the approach from preemptive treatment to prophylaxis in high-risk patients.

## Introduction

Cytomegalovirus (CMV) is a double-stranded DNA virus of the herpesvirus family that establishes latency in the host after primary infection and can reactivate during periods of immunosuppression. CMV infection is the most common clinically significant infection following allogeneic hematopoietic stem cell transplantation (HCT) (1–3). Infection usually results from reactivation in seropositive recipients, and seropositive recipients with seronegative donors are considered to be at the highest risk of infection and disease (4). In the absence of appropriate prevention and timely treatment, CMV causes significant morbidity and mortality following HCT. Beyond the direct effect of CMV disease and the organ dysfunction it causes, CMV has indirect and immune-modulating effects on the host, as manifested by an increased risk of other opportunistic infections (including fungal infections) (5–7), a bidirectional relationship with graft-versus-host disease (GVHD) (8, 9), and increased non-relapse mortality (10). In the absence of prophylaxis, up to 80% of seropositive patients undergoing allogeneic HCT experience CMV reactivation (11).

Over the years, different approaches to CMV infection post-HCT have been adopted, including prophylaxis and preemptive therapy (PET). In this review, we provide an overview of CMV infection in pediatric HCT recipients, focusing on the most recent developments in prevention, risk stratification, and management.

## Epidemiology and risk factors

CMV is a ubiquitous virus (12). Primary infection occurs via various modes of transmission: person-to-person through virus-containing secretions, mother-to-infant transmission, receipt of blood products, and solid organ transplantation or HCT (12–14). After primary infection, the virus establishes a lifelong latency in hematopoietic progenitor cells, blood monocytes, and tissue cells (12, 14).

In the United States, approximately 50% of the population is CMV seropositive, with varying prevalence depending on age, sex, birthplace, and socioeconomic status (15, 16). Approximately 30% of pediatric HCT recipients develop CMV infection between days +30–100 post-HCT (17).

An important risk factor for CMV infection and disease in HCT recipients is the combination of donor and recipient serostatus (18). HCT recipients are at the highest risk when the recipient is seropositive and the donor is seronegative (1, 18). Other risk factors for CMV infection and disease include lymphopenia, immunosuppressive therapy (including steroid use and post-HCT cyclophosphamide) (19), type of transplant, and post-transplant complications (such as GVHD) (1). Recipients of T-cell-depleted umbilical cord blood, matched unrelated, mismatched, and haploidentical transplants are at a higher risk than recipients of matched-related transplants (1, 18).

## Diagnosis and definitions

The initial determination of CMV serostatus in recipients and donors is determined by serologic measurement of CMV-IgG. Currently, post-HCT diagnosis of CMV infection relies on molecular testing to detect CMV DNA in whole blood, serum, and plasma. The definitions of CMV infection and disease are based on consensus by a group of expert panels and are updated regularly for clinical trial purposes. The most recent definitions were published in 2024: *CMV infection* is defined as CMV detection in the blood or CMV DNAemia (CMV DNA detection by qPCR). *Clinically significant CMV infection (csCMVi)* is defined as the development of CMV disease or the need to initiate anti-CMV preemptive therapy for DNAemia above a certain threshold. *CMV disease* is defined as an end-organ disease; that is, CMV infection of a specific organ is demonstrated by the presence of symptoms in the setting of CMV infection and, ideally, documentation of the presence of CMV or CMV-related changes in tissue. End-organ diseases include pneumonia, gastrointestinal disease, hepatitis, central nervous system infection, retinitis, nephritis, cystitis, myocarditis, and pancreatitis. End-organ disease can be classified as proven, probable, or possible, depending on the availability of histological findings and CMV documentation in tissue.

*Refractory CMV infection* is defined as a CMV DNAemia increase of more than 1 log_10_ from the peak viral load or persistence after at least two weeks of appropriate antiviral therapy.

*Refractory CMV end-organ disease* is defined as worsening signs and symptoms, progression to end-organ disease during treatment, or lack of improvement after at least two weeks of appropriate antiviral therapy. *Resistant CMV infection* is defined as refractory CMV infection, in addition to viral genetic alterations that decrease susceptibility to one or more antiviral drugs (20).

## Initial management

### Preemptive therapy and prophylaxis

The initial approaches to CMV infection in HCT recipients consist of PET, antiviral prophylaxis, or a combination of both. PET aims to detect CMV DNAemia early through frequent (usually weekly) monitoring and initiation of antiviral therapy before the development of CMV disease at predefined thresholds. Prophylaxis involves administering antiviral therapy to high-risk patients early in the post-transplant course and throughout the period of the highest risk of CMV reactivation (21).

Historically, antivirals for the management of CMV infection have carried undesirable side effects, such as myelosuppression from ganciclovir and valganciclovir and nephrotoxicity from foscarnet and cidofovir. These toxicity profiles, in the absence of other alternatives and the widespread availability of PCR, frequently favor PET.

In 2017, a phase 3 clinical trial showed that letermovir, a CMV viral terminase complex inhibitor, started prophylactically within 28 days of HCT and continued for up to 100 days post-transplant, resulted in a significant reduction in csCMVi in CMV-seropositive adult allogeneic HCT recipients. Letermovir was accordingly approved by the United States Food and Drug Administration (FDA) as a safe and effective medication for primary CMV prophylaxis in CMV seropositive adult allogeneic HCT recipients in the first 100 days post-transplant (22), and this has since become of the standard of care in this population (1) More recently, the duration of letermovir has been extended to 200 days in higher-risk in higher risk patients based on individualized criteria (23). Studies including adult participants suggest that although PET may prevent development of CMV disease, patients with CMV viremia prompting PET have higher overall and non-relapse related mortality compared to patients with no CMV reactivation and not requiring PET (24). Moreover, one study showed that adult patients on letermovir had lower rates of csCMVi and hospitalization, with no incidence of CMV disease compared to aa ∼5% incidence of CMV disease in patients receiving PET (25).

In 2024, the FDA approval of letermovir was expanded to include pediatric allogenic HCT recipients aged ≥6 months and weighing ≥6 kg. This approval was based on the results of a small phase 2b, single-arm, non-comparative study (NCT03940586). Data from a clinical trial that included 28 adolescents 12–18 years of age demonstrated a favorable safety profile and comparable rates of csCMVi as seen in the pivotal phase 3 adult study: 24% through week 24 post-HCT (26). Until recently, data on the use of letermovir in pediatrics remained anecdotal through its use as salvage therapy in single patients or case series. A recently published meta-analysis of letermovir prophylaxis in pediatric HCT recipients reviewed 12 observational studies, including a pooled population of 253 pediatric HCT recipients (27). Letermovir was given for 92–221 days and the CMV infection rate was 10.9%. No CMV disease was reported during letermovir prophylaxis, and there were no CMV-related deaths. For the four studies that included a control group (using acyclovir or valacyclovir), the incidence of CMV infection was significantly lower in the letermovir group (RR 0.29, 95% CI 0.16–0.56, *p* < 0.001). The rate of letermovir discontinuation was low (2.4%), and adverse events were mild and infrequent, with no reported myelosuppression or nephrotoxicity (27). Dosing was weight-based, and the dose was reduced by 50% in the case of concurrent cyclosporine administration.

Additional articles reporting the use of letermovir in pediatric HCT have been published in 2025. These studies have supported the safety and tolerability of letermovir compared to valganciclovir and ganciclovir (27), provided additional efficacy data in higher-risk umbilical cord and haploidentical HCT recipients, supported early vs. later initiation of letermovir (28), and demonstrated lower cumulative CMV reactivation up to 1-year post-transplant. as well as the potential for delayed immune reconstitution in letermovir recipients (29).

FDA approval for pediatric use, as well as the results of a recent meta-analysis and other publications, highlight the importance and safety of using letermovir prophylaxis in pediatric HCT recipients in the first 100 days post-transplant, in line with the current adult guidelines. The recently published ECIL-10 guidelines for management of CMV in patient undergoing allogeneic stem cell transplant has incorporated this data and recommends considering letermovir prophylaxis in high risk pediatric patients (28). This is expected to be reflected as well in the recommendations of the upcoming updated ASTCT guidelines for CMV prevention in HCT.

### Viral and immune-response monitoring

Concerns regarding letermovir prophylaxis include breakthrough infection and the potential for delayed CMV-specific cellular reconstitution, leading to late-onset CMV, which may be due to decreased CMV antigen exposure (29).

The initial study prompting FDA approval of letermovir demonstrated the potential for breakthrough CMV DNAemia while receiving prophylactic treatment. Consequently, the current guidelines recommend continued CMV monitoring during letermovir prophylaxis. Monitoring is usually recommended for up to 6 months post-HCT, based on the initial letermovir trial showing significant rates of reactivation up to day 180 after discontinuation of letermovir, as supported by other real-life studies suggesting the same. Letermovir prophylaxis and monitoring for CMV DNAemia may be extended in patients assessed to be at high risk of viral reactivation. Monitoring may have to be extended to select patients receiving letermovir prophylaxis for longer periods or those deemed to have risk factors for the later development of csCMVi. The cut-offs for the initiation of PET are institution-dependent and patient-specific. Studies have shown success in preventing the progression to CMV disease using thresholds of 2–3 log_10_ IU/ml (30). One pediatric study performed prior to routine letermovir prophylaxis demonstrated that initiation of PET at ≥1,000 IU/ml (≥3log_10_) vs. <1,000 IU/ml resulted in a higher peak viral load and longer duration of viremia, though without significant differences in end-organ disease, overall survival, or non-relapse-related mortality at 12 months post-HCT (31).

Measuring CMV-specific T-cell-mediated immunity (CMV CMI) has been explored to identify patients at risk for reactivation following discontinuation of prophylaxis and CMV-seropositive or viremic patients at risk of progression to csCMVi. CMV CMI combined with an assessment of patient risk factors may allow for individualized plans for primary and secondary prophylaxis, thresholds for PET, and duration of letermovir prophylaxis (32). This is mostly based on adult studies that explored the role of CMV CMI in stratifying the risk of CMV infection over time through serial monitoring and predicting the progression of DNAemia to csCMVi. Few pediatric studies have explored the role of CMV CMI in predicting the risk of CMV infection and recurrence. Although these studies had small sample sizes, they showed promising results for the clinical use of CMV CMI therapy (33, 34).

Adult studies suggest that letermovir prophylaxis results in delayed development of CMV-specific cellular immunity, with a 100-day delay in the development of CMV-specific immunity and lower CMV-specific CD4 cells noted up to 1-year post-transplant in the letermovir group (29, 35).

## Treatment approaches

### Breakthrough infection on letermovir

Breakthrough CMV reactivation on letermovir can occur even in the absence of known genotypic resistance (36, 37). Breakthrough infection often results in low-level viremia and is typically managed with alternative antiviral therapy if the viral load reaches a predetermined threshold or if CMV disease develops (36). Risk factors for breakthrough infection include higher cumulative steroid exposure, GVHD prophylaxis with post-transplant cyclophosphamide or calcineurin inhibitors in combination with mycophenolate, low-grade CMV replication (21–149 IU/ml) at the time of letermovir initiation or during prophylaxis, and acute GVHD (36, 38).

As noted above, CMV reactivation following letermovir discontinuation has been documented in several studies, occurring in 12%–45% of patients (39–41). Risk factors for late CMV infection after letermovir discontinuation include severe neutropenia on the day of stem cell infusion, HLA-mismatched donors, and CMV-seronegative donors (42, 43).

### Management of resistant and refractory CMV infection

Refractory and resistant CMV infections occur in 19%–39% and 1.7 to14.5% of adult allogeneic HCT recipients, respectively (17, 44, 45). Limited data in pediatric allogeneic HCT recipients suggest resistance in 4%–10% (17). Resistance typically involves mutations in UL97 (conferring resistance to ganciclovir and valganciclovir) or UL54 (potentially conferring resistance to ganciclovir, valganciclovir, foscarnet, and cidofovir) (45).

The initial evaluation and management of refractory CMV infection or end-organ disease involves optimizing the immune status (e.g., decreasing immune suppression) when feasible, ensuring that antiviral therapy is appropriately dosed, and evaluating antiviral drug resistance (44). Alternative antiviral dosing or therapy may be explored if antiviral resistance is detected and/or when testing is pending (44).

Maribavir is a novel antiviral agent that received FDA approval in 2021 for the management of refractory/resistant CMV infection and end-organ disease (46). Maribavir is a benzimidazole antiviral that inhibits multiple stages of CMV replication (47). Maribavir has been associated with a lower treatment effect in patients with higher baseline viral loads, acute graft-versus-host disease, and T-cell depletion (48, 49). Adverse effects include dysgeusia and gastrointestinal distress (e.g., nausea, vomiting, and diarrhea) (46). In the AURORA trial, a multicenter, double-blind, randomized, phase 3 trial comparing maribavir to valganciclovir for first asymptomatic CMV infection in HCT recipients 16 years and older, maribavir showed less neutropenia and discontinuation of therapy and comparable CMV viremia clearance to that of valganciclovir during post-treatment follow-up, although it failed to achieve non-inferiority (49). The SOLSTICE trial, a phase 3, randomized, open-label trial comparing maribavir to investigator assigned therapy (IAT: valganciclovir/ganciclovir, foscarnet, or cidofovir) for the treatment of refractory CMV infection with or without resistance in HCT and solid organ transplant recipients showed superiority of maribavir for CMV viremia clearance and symptom control (50). Similarly, maribavir appears to be as effective as foscarnet for viral clearance, with similar resistance rates (51). Pediatric data are limited, although case reports and series have shown results like those in adults in terms of safety and efficacy (52, 53).

## Future directions

### Virus-specific T-cells (VSTs)

Given the limited antiviral options available, alternative treatment strategies have been investigated. One promising innovation is the use of virus-specific T-cells (VSTs). This therapy involves manufacturing or isolating T-cells that recognize specific viral epitopes, and the cells can be collected from a patient's HCT donor or from a third party (54–56). VSTs are manufactured by stimulating donor peripheral blood mononuclear cells (PBMCs) with virus-specific peptides. T cells are then selectively amplified and expanded in culture (55). Alternatively, T-cells can be isolated from seropositive donors, stimulated with viral peptides, and antigen-specific cells can be isolated (55).

Current research suggests that CMV-specific VSTs are effective in treating CMV viremia and end-organ diseases (55). One study of pediatric allogeneic HCT recipients treated with VSTs for CMV and other double-stranded DNA viruses showed no difference in clinical response between donor-derived and third-party VSTs (54).

### Vaccine

Triplex® is a recombinant attenuated poxvirus vaccine (modified vaccinia Ankara) that expresses immunodominant CMV antigens (57). A multicenter, double-blind, randomized, placebo-controlled phase II trial was performed, which included 102 CMV-seropositive adult allogeneic HCT recipients (57). The study participants received the Triplex vaccine or placebo on days +28 and +56 following HCT. Vaccine recipients showed a reduction in clinically relevant CMV events, higher levels of CMV-specific T-cells in the first 100 days post-HCT, and no vaccine-related deaths, infections, or serious adverse events were observed (57).

Another small study evaluated the feasibility, safety, and immunogenicity of vaccinating matched-related HCT donors with the Triplex vaccine. This phase 1 trial showed that the vaccine was feasible and safe, with no effects on recipient engraftment. Although the vaccine was not ultimately powered to assess efficacy, the results indicated a higher frequency of functionally activated CMV-specific T-cells early post-HCT in pairs with vaccinated donors and continued expansion of the T-cell population during immune reconstitution (58). Phase 1 and 2 studies of CMV-seropositive pediatric allogeneic transplant recipients are ongoing (NCT03354728).

### Monoclonal antibodies

Fistasovimab (NPC-21) is a fully human, neutralizing monoclonal antibody that targets the epitope of CMV glycoprotein B (59). An *in vitro* study has shown a significant anti-CMV effect (60). A phase 2 study in adult renal transplant recipients showed a lower incidence of CMV disease in patients who received the antibody administered monthly as an intramuscular injection, although there was no significant benefit in preventing CMV infection (59).

## Discussion

CMV remains the most common and clinically significant infection in pediatric HCT recipients. Despite significant advancements and ongoing research, additional well-designed, larger pediatric-specific studies are still needed to optimize the diagnosis, prevention, risk stratification, and treatment of CMV infection in this patient population. The approval of letermovir has been the most significant development in the prevention of CMV disease in pediatric HCT recipients in recent years. More long-term longitudinal real-world data on CMV infection and disease during and after discontinuation of letermovir are needed, including studies on immune reconstitution in the context of letermovir prophylaxis.

Additional information on the use of CMV CMI and its integration in clinical practice in the pediatric population is needed, although this seems to hold promise as a good guidance measure in tailoring the need and duration of prophylaxis, as well as the need and frequency of monitoring and threshold for PET in selected patients.

In addition to a growing armamentarium of antiviral drugs with the approval of maribavir in recent years, CMV vaccines, monoclonal antibodies, and VSTs hold hope for additional means to prevent and treat CMV disease and change its landscape in HCT recipients, allowing for continued improved outcomes and reduced mortality.
